# Breastfeeding practices among long-standing refugees in Jordan: insights from a cross-sectional study

**DOI:** 10.3389/fpubh.2025.1644659

**Published:** 2025-08-14

**Authors:** Nuha Qasem, Amjad Al-Shdaifat, Deema Abu-Khader, Sajeda Jaraa, Jelan AlOthman, Lina Shatat, Lara Alsaif

**Affiliations:** ^1^Department of Family Medicine, Radiology, and Emergency Medicine, Faculty of Medicine, Hashemite University, Zarqa, Jordan; ^2^Faculty of Medicine, Hashemite University, Zarqa, Jordan

**Keywords:** exclusive breastfeeding, infant feeding practices, Palestinian refugees, maternal health, breastfeeding determinants, refugee health

## Abstract

Exclusive breastfeeding (EBF) is a crucial public health strategy that reduces infant morbidity and mortality, yet rates remain suboptimal among refugee populations. This study examines breastfeeding practices and determinants of EBF among long-standing Palestinian refugees in Jordan through a cross-sectional survey of 249 mothers at the UNRWA Zarqa Camp Health Center. The prevalence of EBF among infants under 6 months was 38.2%, declining with age. Key predictors included birth order, mode of delivery, labor analgesia, and breastfeeding initiation timing. Mothers who delivered vaginally avoided analgesia, and initiated breastfeeding within the first hour had significantly higher EBF rates. Perceived insufficient milk supply was the primary reason for formula introduction. Breastfed infants experienced fewer acute illnesses, antibiotic use, and allergic conditions. Despite moderately positive maternal attitudes toward breastfeeding, fewer than half of the mothers received breastfeeding education, with no significant association between maternal knowledge and feeding methods. These findings underscore the need for interventions promoting early breastfeeding initiation, reducing unnecessary cesarean sections and analgesia, and strengthening breastfeeding education to improve EBF rates and infant health in refugee settings.

## 1 Introduction

Breastfeeding is regarded as the optimal source of nutrition for infants (1), providing unparalleled health benefits. The World Health Organization and the American Academy of Pediatrics recommend exclusive breastfeeding (EBF) for the first 6 months of life to achieve optimal growth, development, and health (2).

By 2030, the Sustainable Development Goals (SDG) target to end preventable deaths of newborns and children under 5 years of age, with all countries aiming to reduce neonatal and under-five mortality (3). Unfortunately, as of 2022, we are still off-track to achieve the 2030 agenda (4). Globally, infectious diseases – mainly pneumonia, malaria, and diarrhea-accounted for 30% of under-five deaths beyond the neonatal period in 2022 (5). Exclusive breastfeeding is linked in literature with lower incidences of diarrhea, otitis media, UTIs, allergic diseases, and pneumonia in infants (6, 7). Furthermore, EBF promotes effective weight gain, cognitive development (8, 9), and may reduce the risk of chronic diseases such as asthma, allergies, diabetes, and obesity (10). Breastfeeding also offers significant benefits to mothers, including protection against breast cancer, improved birth spacing, and potential protection against ovarian cancer and type 2 diabetes (10).

Despite its proven benefits, the rate of EBF globally is still below the goal set by the World Health Assembly Resolution with the target that at least half of infants should be exclusively breastfed for the first 6 months of life by 2025 (11). In Jordan, the rate of exclusive breastfeeding as reported by the United Nations International Children’s Emergency Fund (UNICEF) in 2017 for infants 0 to 5 months was 25% (12). Common barriers to EBF include the lack of maternal knowledge about EBF and its benefits (13), low maternal confidence in the ability to breastfeed (14), perception of insufficient breastmilk (15), and lack of family and community support for EBF (16). Understanding the factors influencing EBF practices remains critical for designing effective interventions.

In acute and long-term emergencies, infants and young children are particularly vulnerable, where risks of malnutrition, infectious diseases, and mortality are significantly increased for children under 2 who are not breastfed and for infants under 6 months who are not exclusively breastfed (17). Among refugee populations, these vulnerabilities are exacerbated. A systematic review found higher mortality rates among children born to refugee parents, with socio-economic disadvantage and infectious diseases playing significant roles (18). In such contexts, EBF emerges as a critical survival intervention.

By the end of 2023, the United Nations High Commissioner for Refugees (UNHCR) reported 117.3 million forcibly displaced people worldwide, most of them were displaced in countries neighboring their country of origin and live in urban areas outside of camps (19).

Jordan is a lower-middle-income country (12) that hosts the second-highest share of refugees per capita in the world from different nationalities (20). In the middle governorate of Jordan, Zarqa camp—the oldest Palestinian refugee camp established in 1949 — hosts long-standing Palestinian refugees who left Palestine as a result of the 1948 War. While many of these refugees are accommodated in camps, the majority live alongside other Jordanians in cities, towns, and villages, and hold Jordanian citizenship (21).

While government hospitals are the main provider of services to Palestinian refugees in Jordan, UNRWA (United Nations Relief and Works Agency for Palestine Refugees) remains an important healthcare provider particularly for the poorest population, offering mother–child care services to both camp and non-camp residents (21).

Breastfeeding practices are influenced by diverse and multifaceted factors that include medical conditions, hospital protocols, socioeconomic status, cultural beliefs, media and marketing influence, workplace support, and individual factors (22). However, studies that investigated infant feeding practices among long-standing refugees in Jordan are limited. This study aims to fill that gap by investigating breastfeeding and infant feeding practices among infants aged 0–6 months in long-standing Palestinian refugee communities within the middle governorate of Jordan. This research also explores predictors of exclusive breastfeeding and its association with the infant’s health. As such, it may serve as a valuable contribution to the scientific literature on the challenges faced by these vulnerable populations.

## 2 Materials and methods

### 2.1 Study design and setting

A cross-sectional study was conducted using a face-to-face interview with mothers of infants 6 months old or below who attended the UNRWA Zarqa Camp Health Center (ZCHC) during the period June 4th to August 3rd, 2023.

The study outcomes are expected to find the prevalence of breastfeeding among these infants and determine the predictors of exclusive breastfeeding and its association with infant’s health.

All mothers to infants aged 6 months or younger who attended maternal and child health services at UNRWA ZCHC during the period of the survey, and who were willing to participate were recruited.

### 2.2 Sample size

The sample size was calculated based on the following formula: (23).


$$n=\frac{Z^{2}P(1-P)}{d^{2}}$$

Since exclusive breastfeeding for the first 6 months is the recommended feeding practice by all health organizations, we relied on this indicator for calculating the sample size.

Based on the results of a recent study (24) about exclusive breastfeeding among Palestinian refugees in Jordan which included 307 infants aged 6 months or younger in 2017 at UNRWA health centers, the prevalence of exclusive breastfeeding among these infants was 34%. Hence the sample size to achieve a precision of ±5% with a 95% Confidence Interval (CI) was 344, which was not achieved in the time available for data collection according to the timetable of the study, a total of 249 participants were included in this study.

### 2.3 Data collection tools

A validated, constructed, anonymous, and confidential questionnaire was employed to measure different underlying constructs. It was formulated in Arabic and was carefully designed to avoid leading or biased questions. It was filled by the researchers during face-to-face interviews with participating mothers. The interview was initiated by asking the mother to fill a consent form, and a participant information sheet was given to the participant which included explanation about the aim and importance of the study, assuring the safety and privacy of their data, and what to do and whom to contact if they decided to withdraw from the study at any time.

The first part of the questionnaire asked about sociodemographic information like the mother’s age, nationality, level of education, occupation, the family’s place of residence (inside or outside the refugee camp), the source of family income as well as the amount of monthly income (voluntary question), and maternal chronic diseases and smoking status. In addition to information about the infant (age, gender, and birth order).

The next section enquired about the birth conditions (term or preterm, place and mode of delivery, analgesia used, postpartum hemorrhage, and infant’s birth weight).

The following section included questions about maternal height, pre-pregnancy and pre-delivery weight, the timing of the first breastfeeding, skin-to-skin practice, rooming-in during birth hospitalization, and current infant feeding method.

The next question was specific for breastfed babies and asked about breastfeeding practices like frequency of breastfeeding, scheduled or upon request, and signs of infant’s satiety following breastfeeding session.

Then, a section asked about the infant’s diet like the type and reason for introducing milk other than breastmilk to infant’s diet, solid foods, juices and sweets, in addition to questions about signs of sufficient milk intake like the number of daily wet and soiled diapers as well as daily sleep hours.

The following two sections enquired about the mother’s knowledge and attitude toward breastfeeding, and the last section asked about infant’s health issues like attending regular well-child visits, vaccination, number of healthcare visits for acute illnesses, number of episodes of diarrhea and acute respiratory illnesses, use and frequency of antibiotics, hospitalizations, allergic conditions, and infant growth parameters which were recruited from the infant’s health record for the current visit.

### 2.4 Ethical approval

Ethical approval for this study was obtained from the Institutional Review Board Committee of Hashemite University on October 31, 2022, and the Research Review Board Committee of the UNRWA on April 20, 2023.

### 2.5 Data management

The data collected was analyzed using SPSS (Statistical Package for Social Science) version 25. Descriptive statistics such as mean and standard deviation were used for quantitative/continuous data while frequencies and percentages were used for qualitative/categorical data. The chi-square test was employed to assess differences between categorical variables. The independent sample *t*-test and one-way ANOVA were conducted to identify independent determinants. A *p*-value of <0.05 was considered statistically significant with a confidence level of 95%.

## 3 Results

A total of 249 mothers were interviewed and answered the questionnaire. All of them were married. Most of them were younger than 40 years (97.2%), had Jordanian nationality (87.1%), live outside the refugee camp (90%), were housewives (93.2%), had secondary school or lower level of education (79.9%), had four or fewer children (84.3%), were multiparous (75%), had vaginal delivery in their last birth (65.1%), had delivered at hospital (98.4%), and did not receive analgesia during delivery (60.5%). Half of them had normal weight status before pregnancy while 40.2% of them were either overweight or obese, and less than a third of them had normal gestational weight gain during the last pregnancy (31%) (Table 1).

**Table 1:** Sociodemographic, maternal, and infant-related factors and their associations with breastfeeding practices.

Factors		Method of infant feeding				*p*-value
		Breast milk	Formula milk	Mixed	Total	
Maternal age	18–24 years	33a (41.8)	17a (21.5)	29a (36.7)	79 (100)	0.466
	25–30 years	38a (41.3)	12a (13)	42a (45.7)	92 (100)	
	31–39 years	22a (31)	13a (18.3)	36a (50.7)	71 (100)	
	40 years and older	2a (28.6)	2a (28.6)	3a (42.9)	7 (100)	
Nationality	Jordanian	84a (38.9)	40a (18.5)	92a (42.6)	216 (100)	0.101
	Palestinian	8a (30.8)	2a (7.7)	16a (61.5)	26 (100)	
	Syrian	2a (50)	2a (50)	0a (0)	4 (100)	
	Other	0a (0)	0a (0)	2a (100)	2 (100)	
Place of residence	Inside the refugee camp	10a (40)	4a (16)	11a (44)	25 (100)	0.966
	Outside the refugee camp	85a (37.9)	40a (17.9)	99a (44.2)	224 (100)	
Occupation	Housewife	92a (39.7)	38a (16.4)	102a (44)	232 (100)	0.149
	Employee	3a (18.8)	6a (37.5)	7a (43.8)	16 (100)	
	I work from my home	0a (0)	0a (0)	1a (100)	1 (100)	
Level of education	Primary School (Grades 1–6)	4a (44.4)	1a (11.1)	4a (44.4)	9 (100)	0.564
	Middle School (Grades 7–10)	26a (37.7)	14a (20.3)	29a (42)	69 (100)	
	Secondary School (Grades 11–12)	47a (38.8)	22a (18.2)	52a (43)	121 (100)	
	Bachelor’s degree	18a (36.7)	6a (12.2)	25a (51)	49 (100)	
	Master’s degree or higher	0a (0)	1a (100)	0a (0)	1 (100)	
Family income (Jordanian Dinar)	<250 JD	25a (42.4)	10a (16.9)	24a (40.7)	59 (100)	0.636
	250–500 JD	54a (36.7)	25a (17)	68a (46.3)	147 (100)	
	500–750 JD	12a (42.9)	7a (25)	9a (32.1)	28 (100)	
	750–1000 JD	1a (12.5)	2a (25)	5a (62.5)	8 (100)	
	1000–1500 JD	0a (0)	0a (0)	1a (100)	1 (100)	
Infant’s age	Less than 1 week	4a (100)	0a (0)	0a (0)	4 (100)	0.016
	1 week-4 weeks	27a, b (40.3)	5b (7.5)	35a (52.2)	67 (100)	
	1–2 months	11a (32.4)	4a (11.8)	19a (55.9)	34 (100)	
	2–4 months	27a (38.6)	15a (21.4)	28a (40)	70 (100)	
	4–6 months	26a, b (35.1)	20b (27)	28a (37.8)	74 (100)	
Term or preterm	Preterm	10a (25.6)	9a (23.1)	20a (51.3)	39 (100)	0.19
	Term	85a (40.7)	34a (16.3)	90a (43.1)	209 (100)	
Infant’s gender	Male	50a (38.2)	19a (14.5)	62a (47.3)	131 (100)	0.398
	Female	45a (38.5)	24a (20.5)	48a (41)	117 (100)	
Child’s birth order	First	23a (36.5)	14a (22.2)	26a (41.3)	63 (100)	0.043
	Second	30a (56.6)	8a, b (15.1)	15b (28.3)	53 (100)	
	Third	14a (27.5)	8a (15.7)	29a (56.9)	51 (100)	
	Fourth	11a (25.6)	8a (18.6)	24a (55.8)	43 (100)	
	Fifth or beyond	17a (43.6)	6a (15.4)	16a (41)	39 (100)	
Mode of delivery	Unassisted VD	70a (44.3)	23b (14.6)	65a, b (41.1)	158 (100)	0.02
	Assisted VD (vacuum or forceps)	1a (25)	1a (25)	2a (50)	4 (100)	
	Elective CS	5a (12.5)	10b (25)	25b (62.5)	40 (100)	
	Emergency CS	19a (40.4)	10a (21.3)	18a (38.3)	47 (100)	
Analgesia received during delivery	No analgesia	69a (46)	20b (13.3)	61b (40.7)	150 (100)	0.014
	Epidural	3a (30)	1a (10)	6a (60)	10 (100)	
	General anesthesia	18a (26.9)	20b (29.9)	29a, b (43.3)	67 (100)	
	Spinal anesthesia	3a (18.8)	1a (6.3)	12a (75)	16 (100)	
	IM injection analgesia	1a (33.3)	1a (33.3)	1a (33.3)	3 (100)	
	I do not know	1a (50)	1a (50)	0a (0)	2 (100)	
Heavy vaginal bleeding	No	88a (39.1)	39a (17.3)	98a (43.6)	225 (100)	0.738
	Yes	6a (31.6)	3a (15.8)	10a (52.6)	19 (100)	
Maternal pregestational weight status	Underweight	10a (45.5)	5a (22.7)	7a (31.8)	22 (100)	0.518
	Normal weight	45a (39.8)	20a (17.7)	48a (42.5)	113 (100)	
	Overweight	20a (31.3)	13a (20.3)	31a (48.4)	64 (100)	
	Obese	8a (29.6)	3a (11.1)	16a (59.3)	27 (100)	
Category of GWG	Below normal GWG	32a (37.2)	18a (20.9)	36a (41.9)	86 (100)	0.833
	Appropriate GWG	24a (34.3)	13a (18.6)	33a (47.1)	70 (100)	
	Excessive GWG	27a (38.6)	10a (14.3)	33a (47.1)	70 (100)	
Timing of the first breastfeeding	Within the first hour after birth	51a (46.4)	16a (14.5)	43a (39.1)	110 (100)	0.000
	1–3 h after birth	20a (51.3)	1b (2.6)	18a (46.2)	39 (100)	
	3–6 h after birth	7a (43.8)	0a (0)	9a (56.3)	16 (100)	
	6–12 h after birth	4a (40)	1a (10)	5a (50)	10 (100)	
	12–24 h after birth	2a (16.7)	3a (25)	7a (58.3)	12 (100)	
	>24 h after birth	11a (20.4)	15b (27.8)	28b (51.9)	54 (100)	
Skin-to-skin contact within 1st hour of delivery	No	59a (35.1)	30a (17.9)	79a (47)	168 (100)	0.332
	Yes	36a (44.4)	14a (17.3)	31a (38.3)	81 (100)	
Place of delivery	Hospital	91a (37.1)	44a (18)	110a (44.9)	245 (100)	0.159
	House	2a (100)	0a (0)	0a (0)	2 (100)	
	Other	2a (100)	0a (0)	0a (0)	2 (100)	
Who took care of the baby right after delivery?	Normal nursery	32a (34.8)	15a (16.3)	45a (48.9)	92 (100)	0.577
	NICU	18a (35.3)	12a (23.5)	21a (41.2)	51 (100)	
	Rooming-in	45a (42.5)	17a (16)	44a (41.5)	106 (100)	
Level of attitude	Low	18a (48.6)	2a (5.4)	17a (45.9)	37 (100)	0.194
	Moderate	69a (37.3)	35a (18.9)	81a (43.8)	185 (100)	
	High	8a (30.8)	7a (26.9)	11a (42.3)	26 (100)	
Perinatal education about the importance of breastfeeding	No	49a (36.3)	22a (16.3)	64a (47.4)	135 (100)	0.528
	Yes	46a (40.4)	22a (19.3)	46a (40.4)	114 (100)	
Perinatal education about how to practice breastfeeding	No	60a (39)	28a (18.2)	66a (42.9)	154 (100)	0.866
	Yes	35a (36.8)	16a (16.8)	44a (46.3)	95 (100)	
Weaning	No	59a (39.6)	23a (15.4)	67a (45)	149 (100)	0.521
	Yes	36a (36)	21a (21)	43a (43)	100 (100)	

More than one-fourth (27.3%) were smokers, 21 mothers had one or more chronic medical conditions (8.4%) (Table 2). Nineteen mothers (7.8%) had postpartum hemorrhage, 10 of them (4%) had blood transfusions and one mother required surgical intervention to stop bleeding (Table 1).

**Table 2:** Psychological, physical, and economic factors and their association with infant feeding.

Factors		Method of Infant Feeding				*p*-value
		Breast milk	Formula milk	Mixed	Total	
Have you faced psychological crisis when you breastfeed your baby	No	73a (76.8)	31a (70.5)	86a (78.2)	190	0.588
	Yes	22a (23.2)	13a (29.5)	24a (21.8)	59	
Do you have fears or stresses regarding being a refugee	No	86a (90.5)	41a (95.3)	97a (88.2)	224	0.402
	Yes	9a (9.5)	2a (4.7)	13a (11.8)	24	
Do you have any difficulties in getting food or medications?	No	53a (55.8)	23a (52.3)	64a (58.2)	140	0.795
	Yes	42a (44.2)	21a (47.7)	46a (41.8)	109	
Are you a smoker	No	71a (74.7)	29a (65.9)	81a (73.6)	181	0.53
	Yes	24a (25.3)	15a (34.1)	29a (26.4)	68	
Do you have any chronic diseases	No	88a (92.6)	39a (88.6)	101a (91.8)	228	0.727
	Yes	7a (7.4)	5a (11.4)	9a (8.2)	21	
Family income (Jordanian Dinar)	<250 JD	25a (42.4)	10a (16.9)	24a (40.7)	59 (100)	0.636
	250–500 JD	54a (36.7)	25a (17)	68a (46.3)	147 (100)	
	500–750 JD	12a (42.9)	7a (25)	9a (32.1)	28 (100)	
	750–1000 JD	1a (12.5)	2a (25)	5a (62.5)	8 (100)	
	1000–1500 JD	0a (0)	0a (0)	1a (100)	1 (100)	
Is the family income sufficient for you and your family	No	58a (61.1)	23a (52.3)	68a (61.8)	149	0.123
	Yes	32a (33.7)	15a (34.1)	39a (35.5)	86	
	Other	5a, b (5.3)	6b (13.6)	3a (2.7)	14	

Most participants had a monthly income of 500 JDs or below (84.8%), 43.8% reported having difficulties in getting food or medications, and 59.8% mentioned that the family’s monthly income is not sufficient to meet their daily needs (Table 2).

Most of the infants were born at term (84.3%), had normal birth weight (2,500–4,000 gm) (78.2%), 52.8% were male, 44.2% had their first breastfeeding within the first hour after birth, 32.5% had skin-to-skin contact with their mothers within the first hour after birth, and 42.6% had rooming-in with their mothers during the birth hospitalization.

At the time of the survey, the mean age of the infants was 11.3 weeks (median 12, min. 0, max. 29 weeks, SD: 7.985).

The current method of infant feeding was reported to be exclusive breastfeeding, formula feeding, and mixed feeding by 38.2, 17.7, and 44.2%, respectively.

Exclusive breastfeeding was reported by all mothers and by 40% of mothers to infants less than 1 week of age and 1–4 weeks of age, respectively, while almost only one-third of infants aged 4–6 months were exclusively breastfed (35.1%).

Most breastfed babies were fed upon request (77.6%), had at least 6 breastfeeding sessions daily (82.4%) during the week preceding the interview, had signs of satiety after breastfeeding sessions like sleeping and looking calm and relaxed after the end of the feed (71.7 and 95.1%, respectively).

All infants with mixed feeding were given an infant formula. In addition to infant formula, one infant had received cow’s milk powder not intended for infant feeding, and 4 infants (2.4%) received breastmilk from another mother. The additional milk was introduced since birth in 59.2% of the cases. The reason for the introduction of the additional milk was the mother’s feeling of insufficient breastmilk supply as reported by 52.1% of mothers, poor infant growth (15.9%), to reduce the efforts imposed on the mother (31.5%), lack of time for breastfeeding (22.3%), pediatrician advice (19.4%), advice from other health professionals (6.7%), and medical reasons related to the mother (7.2%).

In addition to milk, other foods were introduced into infants’ diets such as vegetables (14.9%), dairy products (17.4%), fruits (19.8%), rice (12%), and sweets (18.9%).

Factors that were significantly associated with exclusive breastfeeding included infant’s age, infant’s birth order, mode of delivery, analgesia used during delivery, and the timing of the first breastfeed after delivery (Table 1).

As the infant gets older breastfeeding rate declined and formula feeding increased, *p* = 0.016. In addition, the second baby was significantly more likely than the first, the third or higher birth orders to be exclusively breastfed, *p* = 0.043.

Mothers who underwent elective CS were significantly less likely to breastfeed their infants exclusively compared to mothers with normal delivery or emergency CS, *p* = 0.02.

Only mothers who did not receive any analgesia during delivery were significantly more likely to exclusively breastfeed their infants, compared to mothers who received any kind of analgesia during delivery, *p* = 0.014.

Infants who had their first breastfeeding beyond the first 12 h of life were significantly less likely to be exclusively breastfed, *p* = 0.000.

Early initiation of breastfeeding (within the first hour after birth) was reported by 44.2% of mothers and was significantly associated with practicing skin-to-skin contact, not receiving any kind of analgesia during birth, and having a vaginal birth, *p* = 0.000 (Table 3).

**Table 3:** Factors associated with early initiation of breastfeeding.

Factors		Timing of first breastfeed				*p*-value
		No breastfeeding	Less than 1 h	More than 1 h	Total	
Mode of delivery	VD	3a (1.9)	101b (62.3)	58a (35.8)	162	0.000
	CS	5a (5.7)	9b (10.3)	73a (83.9)	87	
Maternal age	18–24 years	3a (3.8)	38a (48.1)	38a (48.1)	79	0.508
	25–30 years	2a (2.2)	39a (42.4)	51a (55.4)	92	
	31–39 years	2a (2.8)	29a (40.8)	40a (56.3)	71	
	40 years and older	1a (14.3)	4a (57.1)	2a (28.6)	7	
Place of residence	Inside the refugee camp	0a (0.0)	14a (56)	11a (44)	25	0.339
	Outside the refugee camp	8a (3.6)	96a (42.9)	120a (53.6)	224	
Occupation	Housewife	8a (3.4)	102a (44)	122a (52.6)	232	0.761
	Employee	0a (0.0)	7a (43.8)	9a (56.3)	16	
	I work from my home	0a (0.0)	1a (100)	0a (0.0)	1	
Level of education	Primary School (Grades 1–6)	0a (0.0)	6a (66.7)	3a (33.3)	9	0.316
	Middle School (Grades 7–10)	2a (2.9)	35a (50.7)	32a (46.4)	69	
	Secondary School (Grades 11–12)	6a (5)	52a (43)	63a (52.1)	121	
	Bachelor’s degree	0a (0.0)	17a (34.7)	32a (65.3)	49	
	Master’s degree or higher	0a (0.0)	0a (0.0)	1a (100)	1	
Family income (Jordanian Dinar)	<250	2a (3.4)	32a (54.2)	25a (42.4)	59	0.761
	250–500	5a (3.4)	62a (42.2)	80a (54.4)	147	
	500–750	1a (3.6)	10a (35.7)	17a (60.7)	28	
	750–1000	0a (0.0)	3a (37.5)	5a (62.5)	8	
	1000–1500	0a (0.0)	0a (0.0)	1a (100)	1	
Birth weight	1000-1500g	0a (0.0)	1a (33.3)	2a (66.7)	3	0.423
	1500-2000g	0a (0.0)	1a (25)	3a (75)	4	
	2000–2500	3a (9.1)	9b (27.3)	21a, b (63.6)	33	
	2500–4000	5a (2.6)	92a (47.4)	97a (50)	194	
	4000–4500	0a (0.0)	6a (46.2)	7a (53.8)	13	
	>4500	0a (0.0)	1a (100)	0a (0.0)	1	
Child’s birth order	First	3a (4.8)	21a (33.3)	39a (61.9)	63	0.105
	Second	2a (3.8)	25a (47.2)	26a (49.1)	53	
	Third	1a (2)	30a (58.8)	20a (39.2)	51	
	Fourth	1a (2.3)	13a (30.2)	29a (67.4)	43	
	Fifth or beyond	1a (2.6)	21a (53.8)	17a (43.6)	39	
Received analgesia during delivery	No	2a (1.3)	96b (63.2)	54a (35.5)	152	0.000
	Yes	6a (6.3)	13b (13.5)	77a (80.2)	96	
Heavy vaginal bleeding	No	7a (3.1)	101a (44.9)	117a (52)	225	0.505
	Yes	1a (5.3)	6a (31.6)	12a (63.2)	19	
Term or preterm	Preterm	2a (5.1)	13a (33.3)	24a (61.5)	39	0.305
	Term	6a (2.9)	96a (45.9)	107a (51.2)	209	
Skin-to-skin contact within the 1st hour of delivery	No	7a (4.2)	38b (22.6)	123a (73.2)	168	0.000
	Yes	1a (1.2)	72b (88.9)	8a (9.9)	81	
Place of delivery	Hospital	8a (3.3)	106a (43.3)	131a (53.5)	245	0.274
	House	0a (0)	2a (100)	0a (0)	2	
	Other	0a (0)	2a (100)	0a (0)	2	
Level of attitude	Low	1a (2.7)	20a (54.1)	16a (43.2)	37	0.427
	Moderate	5a (2.7)	78a (42.2)	102a (55.1)	185	
	High	2a (7.7)	12a (46.2)	12a (46.2)	26	

Overall, mothers had a moderately positive attitude toward breastfeeding (mean 2.95, min. 1, max. 4, SD. 0.515), with no significant association between mothers’ attitude and the method of infant feeding, *p* = 0.194 (Table 1).

During antepartum or postpartum periods, less than half of mothers (45.8%) reported receiving education about the importance of breastfeeding, and only 38.2% received education about how to practice breastfeeding. Receiving education about the importance of breastfeeding and how to practice it had no significant association with the method of infant feeding, *p* = 0.528, and 0.866, respectively (Table 1).

Breastfed infants compared to wholly formula-fed infants were significantly less likely to have acute illnesses requiring health care visits twice or more, to receive antibiotics twice or more in their life, and less likely to have allergic conditions, *p* = 0.017, 0.015, and 0.037, respectively (Table 4).

**Table 4:** Infant health conditions and their association with infant feeding.

Health condition		Breastfeeding or only formula, *n* (%)		*p*-value
		Breastfed	Not breastfed	
Acute illness requiring health care visit	0–1	187a (91.7)	35b (79.5)	0.017
	2 or more	17a (8.3)	9b (20.5)	
Allergic conditions	No	176a (86.3)	33a (75)	0.037
	Yes	23a (11.3)	11b (25)	
	Maybe	5a (2.5)	0a (0)	
Used antibiotics	0–1	201a (98)	40b (90.9)	0.015
	2 or more	4a (2)	4b (9)	
Hospital admission	Never	186a (90.7)	37a (84.1)	0.191
	Once or more	19a (9.3)	7a (15.9)	
Diarrhea episodes	Never	135a (66.5)	22a (51.2)	0.057
	Once or more	68a (33.5)	21a (48.8)	
Respiratory illness	Never	134a (65.4)	24a (54.5)	0.176
	Once or more	71a	20a	

## 4 Discussion

In our sample, most families had a limited monthly income of 500 JDs or below, and nearly 60% reported insufficient income to meet daily needs. These socioeconomic challenges may impact access to healthcare, breastfeeding support, and nutritional resources, potentially influencing breastfeeding practices and infant health outcomes.

Globally, the prevalence of EBF among infants under 6 months of age has reached 48% (25), close to achieving the World Health Assembly 2025 target of 50% (11).

However, the rate of EBF in the Middle East and North Africa regions is still far below global trends. In 2019, the reported rate of EBF in these regions was 30.2% (26). Studies suggest that the drivers of these trends include the poor implementation of the Baby-Friendly Hospital Initiative and of the Code of Marketing of Breastmilk Substitutes, which allows formula companies to expand their markets (27), as well as the high prevalence of prelacteal feeds (mostly water-based), which negatively affect breastfeeding practices (28).

The situation in refugee camps mirrors the regional trends. A study performed in refugee camps in Algeria reported 33.3% exclusive or predominant breastfeeding among infants aged less than 6 months (29). This is comparable to what we found in our study; as only 38% of infants younger than 6 months were exclusively breastfed, with a small improvement to the prevalence that was reported by a similar study among long-standing Palestinian refugees in Jordan in 2017; as the prevalence was 34% (24), and even higher than what was reported among the general population in Jordan in 2017 also, where only 26% of infants younger than 6 months were exclusively breastfed (30). This higher prevalence of EBF compared to the general population could be influenced by several factors, including limited access to formula or other supplementary foods due to economic constraints among refugees, and community health initiatives within refugee camps promoting and supporting breastfeeding as a cost-effective and health-protective practice. In addition, the improvement in social and financial status has paradoxically been associated with a decline in the rate of EBF in certain cultural contexts (31).

In this study, most mothers practiced breastfeeding upon infant demand (77.6%) and reported indicators of adequate feeding, such as satiety (95.1%), suggesting a general understanding of infant hunger and satiety cues among breastfeeding mothers.

### 4.1 Exclusive breastfeeding influencing factors

The EBF was significantly associated with the infant’s age, birth order, mode of delivery, analgesia used during delivery, and the timing of the first breastfeed after delivery.

### 4.2 Infant’s age and birth order

EBF declined sharply from 40% in infants less than 4 weeks old to 35.1% by 4–6 months. This trend aligns with global findings where breastfeeding rates often decline as infants age, largely due to the introduction of formula and other foods. This is despite the EBF recommendations for the first 6 months of life by the World Health Organization (WHO) and other health organizations worldwide (2). Perceived insufficient milk supply (52.1%) was the most common reason for introducing formula, highlighting the need for support and education to address this concern, which was also reported in the literature as the major reason for early introduction of solid foods (32).

Several studies suggest children with earlier birth orders are more likely to be breastfed (33, 34). In our results, the second baby was more likely to be exclusively breastfed which may be attributed to previous experience, as breastfeeding self-efficacy was linked in the literature with prior experience (35–37). First-time mothers often perceive more breastfeeding obstacles (35, 36) and report lower self-efficacy during breastfeeding. On the other hand, with increasing birth orders, mothers experience more distractions when feeding their infants. This is supported by a study that included multiparous mothers (38), as well as another study that found a statistically significant higher percentage of EBF continuation when distractions were reduced (39). These reports may explain, in part, our finding that the second baby was more likely to be exclusively breastfed compared to subsequent babies.

### 4.3 Labor method and analgesia use

Mothers who did not receive any kind of analgesia during labor were found more likely to be exclusively breastfeeding. Neuraxial analgesia during labor has been associated with less likelihood of breastfeeding postpartum and thereafter, as reported in the literature (40, 41).

Intramuscular meperidine and morphine are commonly used for labor analgesia. However, Intrapartum pethidine administration may have a detrimental effect on neonatal behavior, reducing infant alertness, suppressing the rooting and sucking reflex, and may shorten breastfeeding duration as well (42).

Also, cesarean birth is associated with decreased breastfeeding initiation and continuation globally (43, 44). The lower rate of breastfeeding initiation and increased difficulties with breastfeeding in women with cesarean section (CS) deliveries may be attributed to a disruption of the hormonal pathway that stimulates lactogenesis (45).

In our findings, mothers who underwent elective CS were significantly less likely to breastfeed their infants exclusively compared to mothers with normal delivery or emergency CS. Similar findings have been reported in the literature; planned c-sections negatively affect breastfeeding initiation and continuation (46, 47), which may be attributed to the timing of planned cesarean sections, which typically occur before the onset of labor, further disrupting the process of lactogenesis.

One contributing factor to the rising number of CS is maternal request, as documented in the literature (48, 49). Encouraging natural birthing practices, minimizing unnecessary interventions, reducing the number of non-medically indicated CS, and developing targeted strategies for women planning cesarean deliveries, starting from the perinatal period and extending into postpartum, could foster the initiation and continuation of breastfeeding.

### 4.4 Early initiation of breastfeeding

The World Health Organization (WHO) recommends early, and uninterrupted skin-to-skin contact between mothers and infants as soon as possible after birth, along with early initiation of breastfeeding (EIBF), within 1 h of birth (50). These practices have been shown to reduce newborn mortality (51) and positively impact the prevalence and duration of exclusive breastfeeding (52, 53). However, achieving this goal can be challenging due to several factors such as maternal pain after CS delivery, exhaustion, suboptimal hospital environment, and insufficient breastfeeding support.

Research also highlights the association between EIBF (within 24 h of birth) and reduced risks of all-cause neonatal mortality among all live births (54). Despite these benefits, global estimates suggest that fewer than half (42%) of all newborns are breastfed within the first hour of birth (55). Reasons for breastfeeding delays included extended recovery time from spinal anesthesia, maternal fatigue, and uncomfortable breastfeeding position due to post-cesarean pain (56).

In our sample, EIBF was reported by less than half of the mothers (44.2%), a figure consistent with findings from studies conducted in India (56, 57). It was significantly associated with practicing skin-to-skin contact, vaginal delivery, and the absence of analgesia during delivery. Moreover, infants who began breastfeeding more than 12 h after birth were significantly less likely to be exclusively breastfed. These findings emphasize the critical window for breastfeeding initiation and underscore the importance of hospital practices and birth interventions in promoting successful breastfeeding initiation and continuation.

### 4.5 Breastfeeding impact on infant health

Our findings revealed that infants fed on breast milk, whether exclusively or partially, were significantly less likely to experience allergic conditions, to have acute illnesses requiring health care visits, and to receive antibiotics compared to formula-fed infants. These findings reaffirm the protective effects of breastfeeding against infections and allergies, consistent with the established benefits of human milk (7, 58).

### 4.6 Maternal education and attitudes regarding breastfeeding

Step 3 of the Baby Friendly Hospital Initiative (BFHI) underscores the need for all pregnant women to be informed about the benefits and techniques of breastfeeding (50). Evidence suggests that comprehensive breastfeeding counseling and support during the prenatal and postpartum periods can significantly improve breastfeeding outcomes (59). Despite that, our data show that less than half of mothers in our sample have received education during antepartum or postpartum periods, about the importance of breastfeeding. Additionally, only 38.2% received education about how to practice breastfeeding. Limited breastfeeding education in this population may reflect resource constraints, healthcare access issues, or gaps in breastfeeding support within the community. Increasing breastfeeding education in these settings could improve the rates of exclusive breastfeeding and better infant health outcomes.

Despite moderately positive maternal attitudes toward breastfeeding, no significant association was found between maternal attitude and infant feeding method. This indicates that external factors, such as healthcare support and socioeconomic constraints, may play a larger role in influencing feeding practices than maternal preferences alone.

### 4.7 Limitations

Data collection relied on self-reported information from mothers, which may be subject to recall bias. In addition, future longitudinal studies could better identify causal relationships between predictors and breastfeeding outcomes. The representativeness of the sample is also a potential limitation, as the sample was recruited from a single UNRWA health center, which may not fully represent the broader population of Palestinian refugees in Jordan or other refugee settings, and the total number of participants was limited because of the tight time frame of the study. Future studies should include larger and more diverse samples to improve generalizability.

## 5 Conclusion

The findings underline the need for comprehensive strategies to promote breastfeeding in these vulnerable situations, including early initiation, addressing concerns about milk insufficiency, supporting natural delivery practices, and improving breastfeeding education during antenatal and postnatal care. Interventions should also consider socioeconomic barriers and cultural factors influencing feeding practices. In addition, EBF indicators should be adopted as part of routine follow-up and quality assurance mechanisms in primary care centers serving refugee populations. This would help translate research into action, enabling health workers to identify at-risk mothers early, offer targeted support, and continuously improve care quality based on real-time data. Future research should explore in-depth the interplay between individual, familial, and systemic factors influencing EBF practices in similar settings globally.

## Data Availability

The raw data supporting the conclusions of this article will be made available by the authors, without undue reservation.
